# An Oral Inoculation Infant Rabbit Model for *Shigella* Infection

**DOI:** 10.1128/mBio.03105-19

**Published:** 2020-01-21

**Authors:** Carole J. Kuehl, Jonathan D. D’Gama, Alyson R. Warr, Matthew K. Waldor

**Affiliations:** a Division of Infectious Diseases, Brigham & Women’s Hospital, Boston, Massachusetts, USA; b Department of Microbiology, Harvard Medical School, Boston, Massachusetts, USA; c Howard Hughes Medical Institute, Boston, Massachusetts, USA; University of Texas Southwestern Medical Center Dallas

**Keywords:** *Shigella*, animal models, bacillary dysentery, host-pathogen interactions, infant rabbit, pathogenesis, shigellosis

## Abstract

*Shigella* species cause diarrheal disease globally. Shigellosis is typically characterized by bloody stools and colitis with mucosal damage and is the leading bacterial cause of diarrheal death worldwide. After the pathogen is orally ingested, it invades and replicates within the colonic epithelium through mechanisms that rely on its type III secretion system (T3SS). Currently, oral infection-based small animal models to study the pathogenesis of shigellosis are lacking. Here, we found that orogastric inoculation of infant rabbits with Shigella flexneri resulted in diarrhea and colonic pathology resembling that found in human shigellosis. Fasting animals prior to S. flexneri inoculation increased the frequency of disease. The pathogen colonized the colon, where both luminal and intraepithelial foci were observed. The intraepithelial foci likely arise through S. flexneri spreading from cell to cell. Robust S. flexneri intestinal colonization, invasion of the colonic epithelium, and epithelial sloughing all required the T3SS as well as IcsA, a factor required for bacterial spreading and adhesion *in vitro*. Expression of the proinflammatory chemokine interleukin 8 (IL-8), detected with *in situ* mRNA labeling, was higher in animals infected with wild-type S. flexneri versus mutant strains deficient in *icsA* or T3SS, suggesting that epithelial invasion promotes expression of this chemokine. Collectively, our findings suggest that oral infection of infant rabbits offers a useful experimental model for studies of the pathogenesis of shigellosis and for testing of new therapeutics.

## Abstract (precis)

*Shigella* species are the leading bacterial cause of diarrheal death globally. The pathogen causes bacillary dysentery, a bloody diarrheal disease characterized by damage to the colonic mucosa and is usually spread through the fecal-oral route. Small animal models of shigellosis that rely on the oral route of infection are lacking. Here, we found that orogastric inoculation of infant rabbits with S. flexneri led to a diarrheal disease and colonic pathology reminiscent of human shigellosis. Diarrhea, intestinal colonization, and pathology in this model were dependent on the S. flexneri type III secretion system and IcsA, canonical *Shigella* virulence factors. Thus, oral infection of infant rabbits offers a feasible model to study the pathogenesis of shigellosis and to develop and test new therapeutics.

## INTRODUCTION

*Shigella* species are Gram-negative, rod-shaped bacteria that cause bacillary dysentery, a severe and often bloody diarrheal disease characterized by inflammatory colitis that can be life-threatening (1). This enteric pathogen, which is spread by the fecal-oral route in humans, does not have an animal reservoir or vector (1). Annually, *Shigella* infections cause tens of millions of diarrhea cases and ∼200,000 deaths (2, 3). It is likely the leading cause of diarrheal mortality worldwide in individuals older than 5 years (2, 3). Most *Shigella* infections are attributable to Shigella flexneri, one of the four *Shigella* species, although in developed nations, the prevalence of Shigella sonnei is higher (4–7).

The pathogen primarily causes colonic pathology that usually includes mucosal ulceration and erosion due to sloughing of epithelial cells and is typically characterized by acute inflammation, with recruitment of neutrophils and plasma cells, congestion of blood vessels, distorted crypt architecture, and hemorrhage (8, 9). While inflammatory responses to *Shigella* invasion of colonic epithelial cells were thought to be the underlying cause of epithelial cell destruction and hemorrhage, recent evidence suggests that pathogen-mediated destruction of epithelial cells also plays a role in the development of pathology (10).

*Shigella* pathogenesis is attributable to a multifaceted set of virulence factors that enable the pathogen to invade and proliferate within the cytoplasm of colonic epithelial cells and evade host immune responses. The pathogen can also infect and rapidly kill macrophages (11). Most known virulence factors are encoded on a large (>200-kbp) virulence plasmid, which is required for *Shigella* pathogenicity (12–14). Key virulence determinants include a type III secretion system (T3SS) and its suite of protein effectors that are injected into host cells (15) and the cell surface protein IcsA, which directs polymerization of host actin and enables intracellular movement (16, 17). The force generated by intracellular actin-based motility allows the pathogen to form membrane protrusions into neighboring uninfected cells, which the pathogen subsequently enters. Cell-to-cell spread is thought to promote pathogen proliferation in the intestine and evasion of immune cells (11). The ∼30 T3SS effector proteins encoded by genes of the *Shigella* strains have varied functions, but primary roles include facilitating invasion of epithelial cells and suppression of host immune responses, including cytokine production.

Among animals used to model infection, only nonhuman primates develop shigellosis from oral inoculation (18); however, the expense of this model limits its utility. Several small animal models of *Shigella* infection have been developed, yet none capture all the features of natural human infection. Historically, the Sereny test was used to identify *Shigella* virulence factors required for induction of an inflammatory response (19); however, this ocular model bears little resemblance to natural infection. The adult rabbit ligated ileal loop model has proven useful for the study of *Shigella* virulence factors (20). However, this model bypasses the normal route of infection and challenges the small intestine, which is not the primary site of pathology in human infections. Intrarectal guinea pig infection induces colonic pathology and bloody diarrhea (21) and has been used to dissect the contribution of *Shigella* and host factors in several aspects of pathogenesis (22–24). Adult mice, the most genetically tractable mammalian model organism, are recalcitrant to developing disease when inoculated orally (25). As an alternative to oral inoculation, an adult mouse pulmonary model of *Shigella* infection involving intranasal inoculation of mice with *Shigella* has been developed (26); this model provides a platform to investigate host immune responses and vaccine candidates (27, 28), and this model has improved understanding of the innate immune response to *Shigella* infection (29). In contrast to adult mice, infant mice are susceptible to oral inoculation within a narrow window of time after birth, and inoculation with a high dose of the pathogen leads to mortality within a few hours; however, pathology is evident in the proximal small intestine rather than the distal small intestine or colon, and infected suckling mice do not develop diarrhea or intestinal fluid accumulation (30, 31). A zebrafish larva model, in which the *Shigella* T3SS is required for pathogen virulence, has been useful for characterizing cell-mediated innate immune responses to *Shigella* due to the ability to image infection *in vivo* (32, 33). Recently, an infant rabbit intrarectal inoculation model in which animals develop disease and rectal pathology reminiscent of natural infections was described (10). The lack of a robust, oral inoculation-based, small animal model of shigellosis has limited understanding of the role of virulence factors in pathogenesis, particularly of the importance of such factors for enabling intestinal colonization and for generating pathology and clinical signs.

Here, we found that orogastric inoculation of infant rabbits with S. flexneri results in severe disease resembling human shigellosis. Orally infected animals develop diarrhea and colonic pathology marked by damage to the epithelial cell layer and edema. Furthermore, the pathogen invaded and appeared to spread between colonic epithelial cells. We found that both the T3SS and IcsA were required for signs of disease, intestinal colonization, and pathology. In addition, invasion of the pathogen into the epithelial cell layer was required for induction of host interleukin 8 (IL-8) expression. *In situ* mRNA labeling revealed that induction of IL-8 transcripts occurs primarily in cells adjacent to invaded epithelial cells, and not in the infected cells. Thus, our findings suggest that the orogastric infant rabbit model provides a powerful and accessible small animal model for further investigation of factors contributing to *Shigella* pathogenesis and for testing new therapeutics.

## RESULTS

### Infant rabbits develop diarrhea after orogastric inoculation with S. flexneri.

In previous work, we found that orogastric inoculation of infant rabbits with enterohemorrhagic Escherichia coli (EHEC), Vibrio cholerae, and Vibrio parahaemolyticus (34–36) leads to diarrheal diseases and pathologies that mimic their respective human counterparts. Here, we explored the suitability of orogastric inoculation of infant rabbits to model *Shigella* infection. S. flexneri 2a strain 2457T, a human isolate that is widely used in the research community as well as in challenge studies in humans (37), was used in this work. We utilized a streptomycin-resistant derivative of this strain for infections to facilitate enumeration of pathogen CFU (CFU in samples from the rabbit intestine). This strain, which contains a point mutation resulting in a K43R mutation in the small (30S) ribosomal subunit protein RpsL, retains the full virulence plasmid and grows as well as the parent strain.

**FIG 1:**
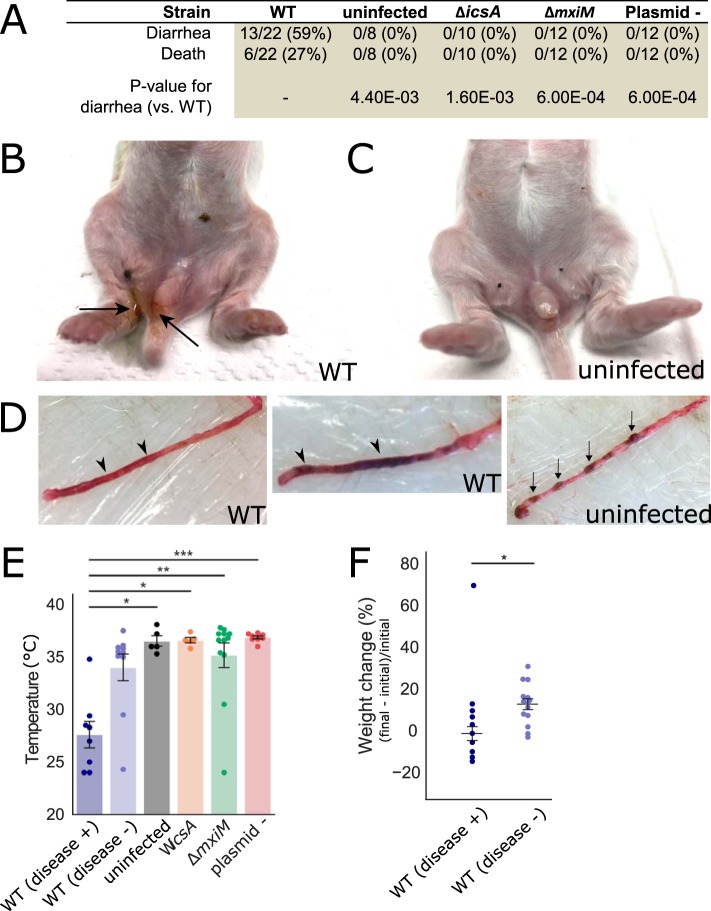
Clinical signs and gross pathology of infant rabbits following orogastric inoculation of S. flexneri. (A) Clinical signs in infant rabbits infected with S. flexneri or isogenic mutant strains. Statistical significance for development of diarrhea between the animals in the WT group and in each of the other groups (uninfected, Δ*icsA*, Δ*mxiM*, and plasmidless [Plasmid -]) was determined using a Fisher's exact test. (B and C) Hind regions of animals inoculated with the WT strain (B) or an uninfected animal (C). Black arrows indicate liquid feces stuck on anus and hind paws. (D) Colons from animals inoculated with the WT strain (left) or of an uninfected animal (right). Arrowheads point to regions of liquid feces, and arrows indicate solid fecal pellets. (E) Body temperature of animals inoculated with the indicated strains 36 hpi or when they became moribund. Standard error of the mean values (error bars) are superimposed. Disease + or − indicates whether or not animals developed diarrhea or became moribund early; all groups were compared to the WT (diarrhea +) group using a Kruskal-Wallis test with Dunn’s multiple-comparison posttest. Values that are significantly different are indicated by bars and asterisks as follows: *, *P* < 0.05; **, *P* < 0.01; ***, *P* < 0.001. (F) Percentage change in weight of infant rabbits infected with the WT strain, grouped by whether or not they developed disease (+ or -). Percentage change in weight is calculated as difference between the final weight of the animal at 36 hpi or the last weight measurement taken when they became moribund (final) and the initial weight of the animal upon arrival in the animal facility (initial). Means and standard errors of the mean values are superimposed. Groups were compared with a Mann-Whitney U test. *, *P* < 0.05.

**FIG 2:**
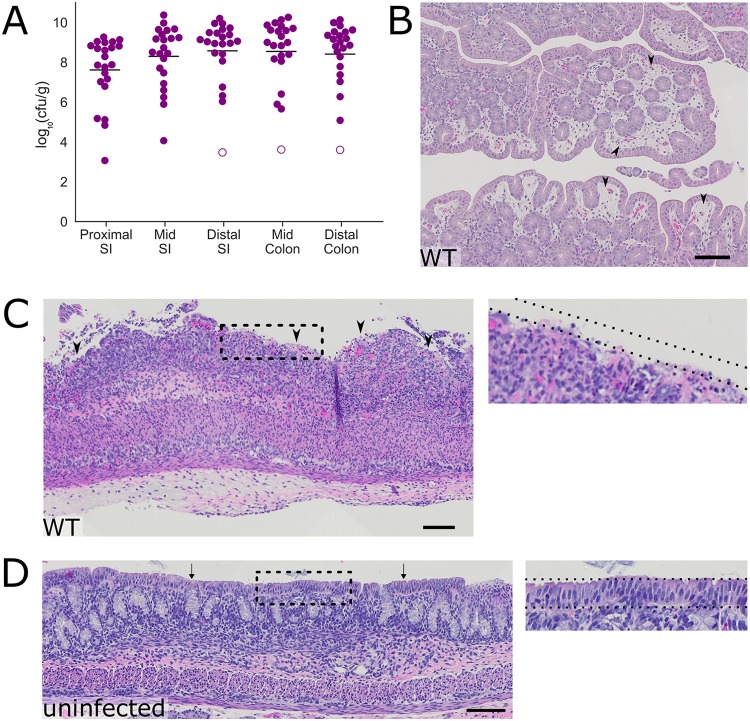
Intestinal colonization and colonic pathology in infant rabbits infected with S. flexneri. (A) Bacterial burden of S. flexneri in the indicated intestinal sections in the small intestine (SI) or colon 36 hpi. Each symbol represents a measurement from one rabbit. Data were plotted as log-transformed CFU (CFU per gram of tissue) (mean values are indicated with bars). Open circles represent the limit of detection of the assay and are shown for animals where no CFU were recovered. (B to D) Representative hematoxylin-and-eosin-stained colonic sections from infected animals (B and C) 36 hpi or uninfected animals (D). Black arrowheads in panel B indicate areas of edema in the lamina propria. Arrowheads in panel C indicate areas where the epithelial cell layer is absent. Arrows in panel D point to the intact layer of epithelial cells seen in the colon. The dashed lines indicate the presence (inset in panel D) or absence (inset in panel C) of the epithelial cell layer. Bars, 100 μm.

In order to investigate infant rabbits as a potential *Shigella* host, we orally inoculated 2- to 3-day-old rabbits that were cohoused with their dam and then monitored for signs of disease. There was considerable variability in the development of diarrhea and colonization in initial studies using suckling rabbits fed *ad libitum*. Previous work using 4-week-old rabbits suggested that a milk component could protect animals from disease by degrading the *Shigella* T3SS components (38, 39); consequently, additional experiments were performed with infant rabbits separated from their lactating dam for 24 h prior to inoculation. Using this protocol, we obtained more reliable clinical disease and robust intestinal colonization. By 36 h postinfection (hpi), the majority (59%) of animals developed diarrhea, which was grossly visible as liquid fecal material adhering to the fur of the hind region of the rabbits (Fig. 1A to C), and high levels of intestinal colonization (see Fig. 2A); occasionally the diarrhea was frankly bloody. We chose the 36 hpi time point because from preliminary time course experiments, we observed that all animals that were going to develop diarrhea developed disease by this time point, and there was significant intestinal pathology at this time. Upon necropsy, the colons of infected animals were often bloody and contained liquid fecal material, in contrast to those of uninfected animals, which contained solid fecal pellets (Fig. 1D). Furthermore, some infected rabbits (27%) succumbed to infection rapidly and became moribund prior to 36 hpi, though not all of these animals developed diarrhea (Fig. 1A). Infected animals had highest bacterial burdens in the colon as well as the mid and distal small intestine (Fig. 2A). The development of disease was associated with higher pathogen burdens in the colon (see Fig. S1A in the supplemental material). Separation of kits from the dam prior to inoculation led to a statistically significant elevation in intestinal colonization (Fig. S1B).

10.1128/mBio.03105-19.1 FIG S1 Factors influencing development of diarrheal disease in infant rabbits after orogastric inoculation of S. flexneri. (A and B) Bacterial burden of S. flexneri in the indicated intestinal sections (small intestine [SI] or colon) at 36 hpi. Each point represents a measurement from one rabbit. Data are plotted as log-transformed colony-forming units (CFU) per gram of tissue. Means and standard error of the mean values are superimposed. Open circles represent the limit of detection of the assay and are shown for animals where no CFU were recovered. For each anatomical section, groups were compared with a Mann-Whitney U test. *P* values: <0.05, *; <0.01, **; <0.001, ***. (A) “disease +” refers to animals infected with the WT S. flexneri strain that developed disease (diarrhea or became moribund early), while “disease -” refers to animals separated from dams for 24 h prior to inoculation refers to animals infected with the WT S. flexneri strain that did not develop disease. (B) “unfasted” refers to animals fed *ad libitum* prior to inoculation, and “fasted” refers to animals separated from dams for 24 h s prior to inoculation. (C) Initial body weights of infant rabbits inoculated with WT S. flexneri, grouped based on whether animal developed disease (diarrhea or early mortality). Means and standard error of the mean values are superimposed. Groups were compared with a Mann-Whitney U test. Download FIG S1, EPS file, 0.3 MB. Copyright © 2020 Kuehl et al. 2020 Kuehl et al. This content is distributed under the terms of the Creative Commons Attribution 4.0 International license.

Although not all S. flexneri-inoculated animals developed signs of disease, infected rabbits that developed diarrhea or died early displayed additional disease signs. The animals that developed disease had significantly lower body temperatures than uninfected animals (8–9°C lower than uninfected animals [Fig. 1E]), and they had significantly smaller gains in body weight than infected animals without disease (−2% versus 13%) (Fig. 1F) over the course of the experiment. Despite the relatively large intra- and interlitter variation in body weight, with a constant pathogen dose per animal (1 × 10^9^ CFU), a lower initial body weight did not appear to be a risk factor for the development of disease (Fig. S1C).

Histopathological examination of the intestines from infected rabbits revealed colonic pathology reminiscent of some of the features observed in infected human tissue, including substantial edema (Fig. 2B) as well as sloughing of colonic epithelial cells (Fig. 2C). In unusual cases, there was massive hemorrhage in the colonic tissue of infected rabbits (Fig. S2). Uninfected rabbits, which were similarly treated (separated from dams for 24 h prior to inoculation [fasted]), did not display colonic pathology and had no edema or disruption of the surface layer of epithelial cells (Fig. 2D). Notably, although the bacterial burden in the colon was similar to that of the distal small intestine (Fig. 2A), substantial pathology was not observed in the distal small intestine, suggesting that organ-specific host factors influence the development of intestinal pathology.

10.1128/mBio.03105-19.2 FIG S2 Examples of severe colonic pathology in infant rabbits inoculated with S. flexneri infection. (A and B) Hematoxylin-and-eosin-stained colonic sections of severe hemorrhage in lamina propria and colonic lumen from animals infected with the WT strain at 36 hpi. Arrowheads in panel A indicate either an area of hemorrhage in the lamina propria or hemorrhage spreading to the lumen (inset in panel A). (B) Hemorrhage and epithelial cell sloughing in colonic lumen. Scale bar, 100 μm. Download FIG S2, TIF file, 2.1 MB. Copyright © 2020 Kuehl et al. 2020 Kuehl et al. This content is distributed under the terms of the Creative Commons Attribution 4.0 International license.

### S. flexneri invades colonic epithelial cells after orogastric infection.

**FIG 3:**
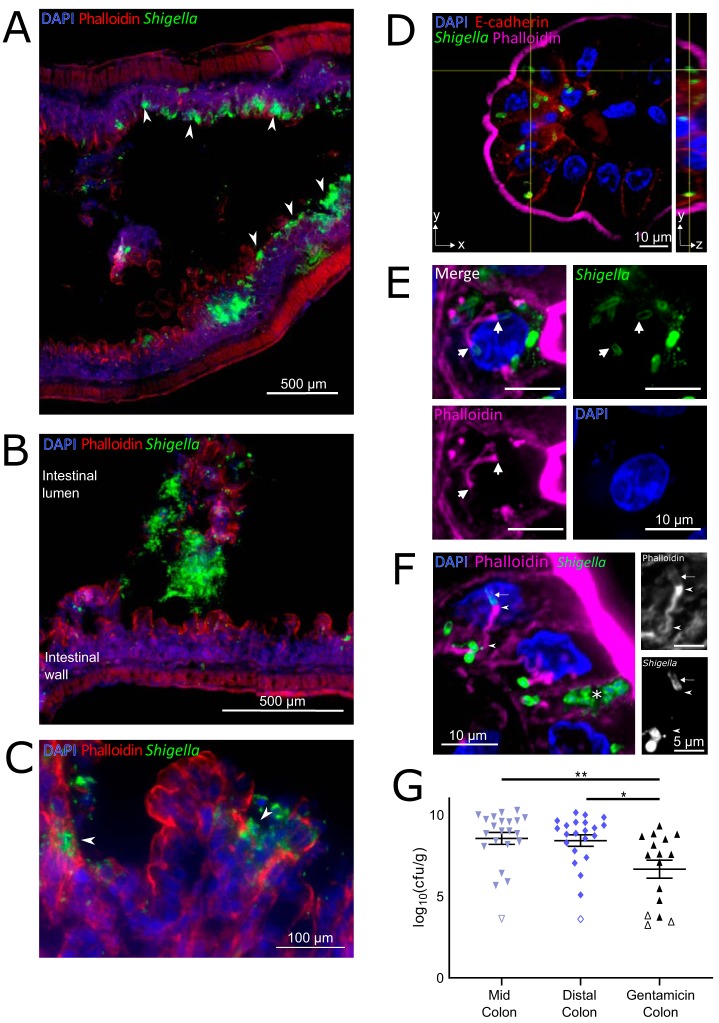
Localization of S. flexneri in the colons of infected infant rabbits. (A to F) Immunofluorescence micrographs of S. flexneri in colonic tissue of infected rabbits 36 hpi. (A) S. flexneri bacteria were found in large numbers in epithelial foci (white arrowheads point to selected foci). (B) S. flexneri bacteria in the lumen of the colon. The intestinal lumen and intestinal wall are labeled. (C) Arrowheads show infection foci where multiple neighboring cells contain intracellular S. flexneri. (D) Immunofluorescence *z*-stack micrograph of S. flexneri within colonic epithelial cells. The left (square) panel shows the *xy* plane at a single *z* position, indicated by the horizontal axis of the cross-hairs in the *yz* projection. The right (rectangular) panel shows *yz* projection along the plane indicated by the vertical axis of the cross-hairs in the *xy* plane. (E) Immunofluorescence micrograph of S. flexneri associated with actin tails within colonic epithelial cells. White arrows point to poles of S. flexneri bacterial cells from which the actin tail is formed. Bars, 10 μm. (F) Immunofluorescence micrograph of S. flexneri forming protrusions during cell-to-cell spread between colonic epithelial cells. The white asterisk marks a likely primary infected cell. Panels show zoomed region of phalloidin or anti-*Shigella* channels. Arrow points to actin surrounding the bacterial cell in a protrusion, arrowheads indicate the actin tail and actin cytoskeleton inside the protrusion at the pole of the bacterial cell and at the base of the protrusion. DAPI (blue), FITC-conjugated anti-*Shigella* antibody (green), phalloidin-Alexa Fluor 568 (red in panels A and C or magenta in panels D to F), and when present, anti-E-cadherin (red in panel D). (G) Bacterial burden of S. flexneri WT strain in the indicated intestinal sections 36 hpi. Each symbol represents the measurement from one rabbit. Data are plotted as log-transformed CFU (CFU per gram of tissue) (means and standard error of the mean values are superimposed). Open symbols represent the limit of detection of the assay and are shown for animals where no CFU were recovered. Statistical significance was determined with a Kruskal-Wallis test with Dunn’s multiple-comparison posttest. *, *P* < 0.05; **, *P* < 0.01.

Tissue sections from the colons of infected rabbits were examined with immunofluorescence microscopy to determine the spatial distribution of S. flexneri in this organ. The pathogen, which was labeled with an anti-*Shigella* antibody, was detected in the intestinal lumen and in many scattered foci within the epithelium (Fig. 3A and B). At low magnification, the signal from the immunostained pathogen appeared to overlap with epithelial cells (Fig. 3A to C). At high magnification, immunostained S. flexneri bacteria clearly evident within the boundaries of epithelial cells, which were visualized with phalloidin staining of actin and an antibody against E-cadherin (Fig. 3D). Several S. flexneri cells were frequently observed within an infected epithelial cell. In some infected epithelial cells, we observed S. flexneri cells associated with phalloidin-stained actin tails (Fig. 3E), and in other foci, we observed S. flexneri in protrusions emanating from a primary infected cell with many cytosolic bacteria (Fig. 3F, asterisk), similar to structures seen in *Shigella* infections of tissue cultured cells (40, 41). The detection of actin tails and protrusions supports the hypothesis that the pathogen is actively spreading within the epithelial cell layer in the colon. S. flexneri cells were primarily localized to the epithelial cell layer and were infrequently observed in the lamina propria, the region of the intestinal wall directly below the epithelial cell layer. We did not find bacteria in the deeper layers of the intestine (Fig. 3A). Hence, following orogastric inoculation of infant rabbits with S. flexneri, the pathogen appears to proliferate both within the colonic lumen and in epithelial cells without penetration into deeper tissues.

We also measured the burden of intracellular S. flexneri in the colon using a modified gentamicin protection assay previously used to study the intracellular burden of Listeria monocytogenes and Salmonella enterica serovar Typhimurium in murine intestinal tissues (42–46). After dissecting intestines from infected infant rabbits, colonic tissue was incubated with gentamicin, an antibiotic that selectively kills extracellular (i.e., luminal) bacteria. We observed an ∼2-log-unit decrease in bacterial burden after gentamicin treatment (Fig. 3G), suggesting that only a small portion of S. flexneri in the colon are intracellular.

### IL-8 transcripts are often observed in epithelial cells near infected cells.

**FIG 4:**
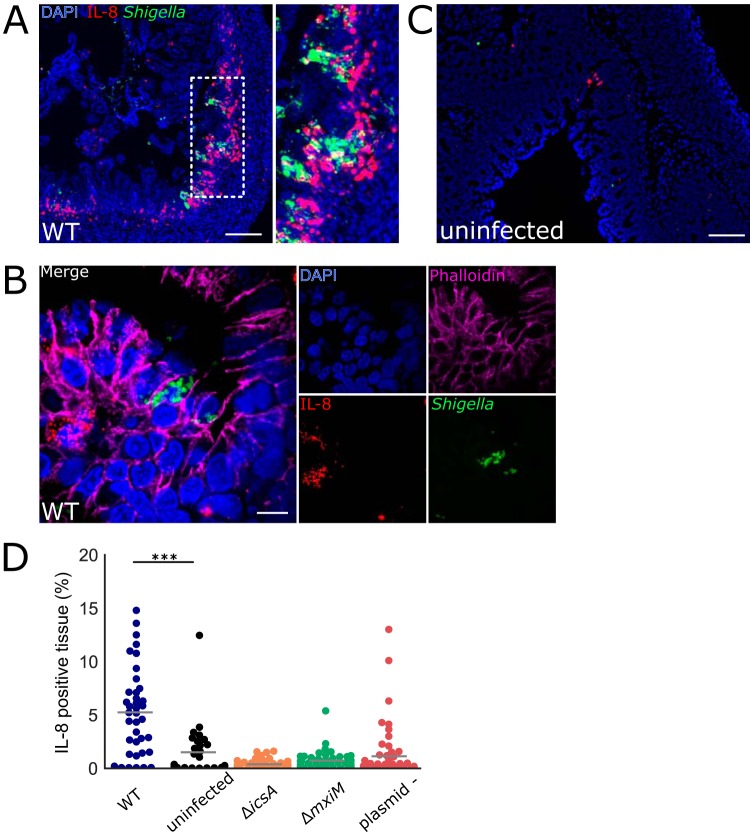
Colonic IL-8 mRNA in rabbits infected with S. flexneri. (A to C) Immunofluorescence micrographs of colonic sections from infant rabbits infected with WT S. flexneri (A and B) or uninfected control (C). Sections were stained with an RNAscope probe to rabbit IL-8 (red), an antibody to *Shigella* (FITC-conjugated anti-*Shigella* green), and with DAPI (blue). (A) Colon section infected with WT S. flexneri. The inset to the right in panel A depicts a magnified view of the boxed area in the left image. Bar, 200 μm. (B) High magnification of colonic epithelium infected with WT S. flexneri. Sections were also stained with anti-E-cadherin antibody (magenta). Bar, 10 μm. (C) Uninfected colon section. Bar, 100 μm. (D) Percentage of IL-8-expressing cells in each field of view from colonic tissue sections stained with probe to rabbit IL-8 from rabbits infected with the indicated strain. See Materials and Methods for additional information regarding the determination of these measurements. Mean values are indicated with bars. All groups were compared to the sections from the uninfected animals. Statistical significance was determined using a Kruskal-Wallis test with Dunn’s multiple-comparison posttest. ***, *P* < 0.001.

We next investigated aspects of the infant rabbit host innate immune response to S. flexneri infection. IL-8, a proinflammatory CXC [chemokine (C-X-C motif)] family chemokine that recruits neutrophils (47), has been shown to be elevated during *Shigella* infection in animal models (10, 21, 48) and in humans (49, 50). However, in preliminary experiments, it was difficult to detect significant elevations of IL-8 transcripts in bulk colonic tissue using a quantitative PCR (qPCR)-based assay. Due to the patchiness of the infection foci observed through immunofluorescence imaging of colonic tissue, we wondered whether a localized response to infection might be masked when analyzing bulk intestinal tissue specimens. Local expression of IL-8 mRNA in S. flexneri-infected tissue was assessed using RNAscope technology, a sensitive, high-resolution *in situ* mRNA imaging platform that permits spatial analysis of mRNA expression. We detected localized expression of IL-8 mRNA in colonic epithelial cells near infection foci in the colon (Fig. 4A and B). In contrast, very few IL-8 transcripts were detected in the colons of uninfected kits (Fig. 4C and D). Combined detection of IL-8 and S. flexneri demonstrated that IL-8-expressing cells were typically near cells containing S. flexneri, but not themselves infected with the pathogen (Fig. 4A, B, and D and Fig. S3). The majority (>90%) of infected epithelial cells did not express IL-8 mRNA, while >40% of these infected cells were adjacent to uninfected cells that did express IL-8 mRNA. Several T3SS effectors from S. flexneri, e.g., IpgD (51) and OspF (52), have been shown to reduce IL-8 expression in infected cells, which may explain the weak or absent IL-8 production in infected cells. There was a wide range in the prevalence of IL-8-producing cells in infected animals (Fig. 4D and Fig. S3). The variability of IL-8 expression after infection may reflect the patchiness of S. flexneri invasion along the colon (Fig. 3A). Together, these observations suggest that S. flexneri infection induces IL-8 mRNA expression (and perhaps additional cytokines as well) in infant rabbits.

10.1128/mBio.03105-19.3 FIG S3 Range of IL-8 expression in colons of infected infant rabbits. (Top left, plot) Percentage of IL-8-expressing cells in each field of view from colonic tissue sections stained with probe to rabbit IL-8 from individual rabbits infected with the WT strain or from uninfected rabbits. Colored dots correspond to micrographs with similar colored borders. Mean values are indicated with bars. (Micrographs) Immunofluorescence micrographs of colonic sections from uninfected animals or infant rabbits infected with WT S. flexneri strain. Sections were stained with a RNAscope probe to rabbit IL-8 (red) and an antibody to *Shigella* (green) and with DAPI (blue). Download FIG S3, TIF file, 2.8 MB. Copyright © 2020 Kuehl et al. 2020 Kuehl et al. This content is distributed under the terms of the Creative Commons Attribution 4.0 International license.

### Narrow bottleneck to *Shigella* infection of the infant rabbit colon.

We attempted to use transposon insertion sequencing (TIS) to identify genetic loci contributing to S. flexneri colonization and pathogenesis, as we have done with V. cholerae (53, 54), V. parahaemolyticus (55), and EHEC (56). Initially, a high-density transposon mutant library in S. flexneri was created using a *mariner*-based transposon that inserts at TA dinucleotide sites in the genome. The library included insertions across the entirety of the genome, including the virulence plasmid (see Table S1 in the supplemental material). Infant rabbits were inoculated with the transposon library, and transposon mutants that persisted after 36 hpi were recovered from the colon. Comparison of the frequencies of insertions in the input and output libraries revealed that the output transposon libraries recovered from rabbit colons contained only ∼20% of the transposon mutants that were present in the input library. These observations suggest that there is a very narrow bottleneck for S. flexneri infection in rabbits, leading to large, random losses of diversity in the input library. These random losses of mutants confound interpretation of these experiments and preclude accurate identification of genes subject to *in vivo* selection. Modifications to the *in vivo* TIS protocol will be necessary to apply TIS to identify additional S. flexneri colonization factors.

10.1128/mBio.03105-19.5 TABLE S1 Transposon library in Shigella flexneri 2a strain 2457T. Download Table S1, XLSX file, 0.01 MB. Copyright © 2020 Kuehl et al. 2020 Kuehl et al. This content is distributed under the terms of the Creative Commons Attribution 4.0 International license.

### Canonical S. flexneri virulence factors are required for intestinal colonization and pathogenesis.

Next, we investigated the requirement for canonical *Shigella* virulence factors in intestinal colonization and disease pathogenesis. First, we tested a strain that lacked the entire virulence plasmid, which contains most of the known virulence factors encoded in the S. flexneri genome, including the T3SS. As anticipated, this strain was avirulent; animals inoculated with the plasmidless (plasmid -) S. flexneri strain did not die or develop diarrhea or reduced temperature (Fig. 1A and E). We also tested isogenic mutants that lack one of two key virulence factors: IcsA (Δ*icsA* strain), which is required for intracellular actin-based motility and cell-to-cell spreading, and MxiM (Δ*mxiM* strain), which is a T3SS structural component (57). *mxiM* deletion mutants do not assemble a functional T3SS, do not secrete T3SS effectors, and do not invade tissue-cultured epithelial cells (57–59). Like the plasmidless strain, the Δ*icsA* and Δ*mxiM* strains did not cause disease; none of the rabbits infected with either of these two mutant strains developed diarrhea, succumbed to infection, or had a reduction in body temperature (Fig. 1A and E). Additionally, none of the mutants induced colonic edema or epithelial cell sloughing, pathological features that characterized wild-type (WT) infection (Fig. 2; see Fig. 6). Collectively, these data indicate that both IcsA and the T3SS are required for *Shigella* pathogenesis in the infant rabbit model.

**FIG 5:**
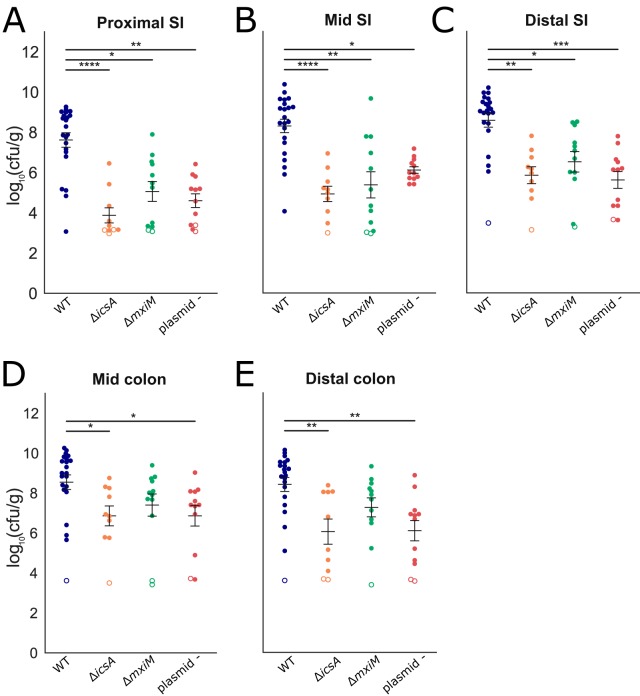
Intestinal colonization of WT and mutant S. flexneri. (A to E) Bacterial burden of the indicated strains in the indicated intestinal sections (small intestine [SI] or colon) 36 hpi. Each symbol represents a measurement from one rabbit. Data are plotted as log-transformed CFU per gram of tissue. Means ± standard error of the mean values are superimposed. Open symbols represent the limit of detection of the assay and are shown for animals where no CFU were recovered. For each section, burdens from all strains were compared to each other; statistical significance was determined using a Kruskal-Wallis test with Dunn’s multiple-comparison. *, *P* < 0.05; **, *P* < 0.01; ***, *P* < 0.001; ****, *P* < 0.0001.

All three of the mutant strains had reduced capacities to colonize the infant rabbit intestine (Fig. 5). Notably, the reduction in the colonization of the *icsA* mutant was at least as great as the other two mutant strains, suggesting that cell-to-cell spreading or the adhesin function of IcsA is critical for intestinal colonization. The colonization defects were most pronounced in the small intestine, where up to 10^4^-fold reductions in recoverable S. flexneri CFU were observed (Fig. 5). Reductions in the colon were less marked and did not reach statistical significance for the Δ*mxiM* strain (Fig. 5).

**FIG 6:**
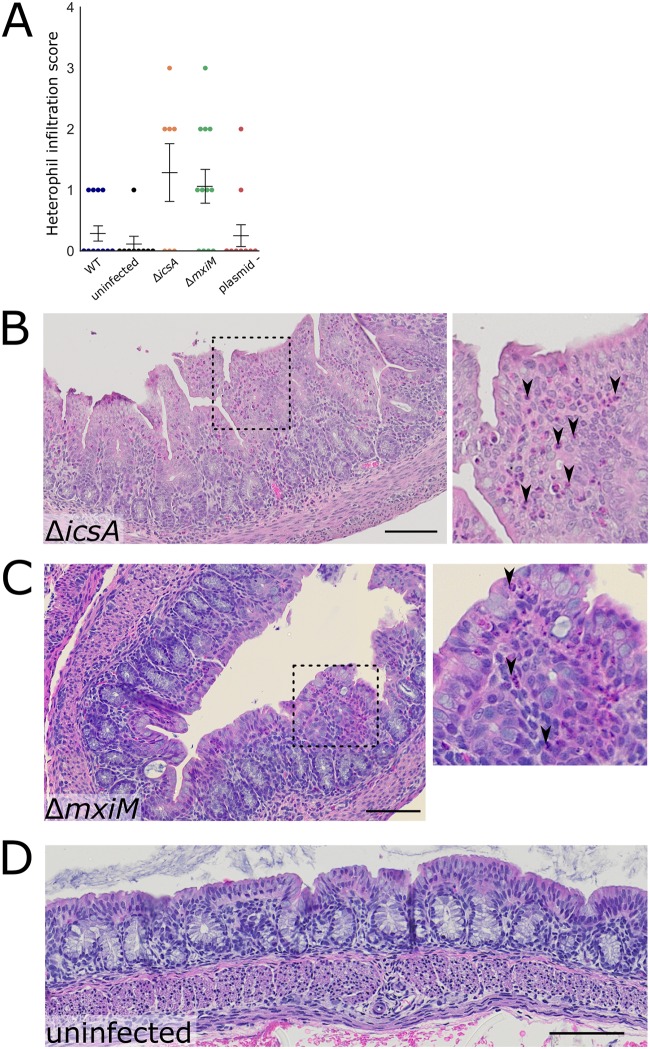
Colonic pathology in rabbits infected with WT or mutant S. flexneri. (A) Histopathological scores of heterophil infiltration in colonic sections of animals infected with indicated strains of S. flexneri. Means ± standard error of the mean values are superimposed. Statistical significance was determined using a Kruskal-Wallis test with Dunn’s multiple-comparison posttest; comparisons that are nonsignificant are not labeled. (B to D) Representative hematoxylin-and-eosin-stained colonic sections from rabbits infected with the indicated strains 36 hpi. In panel B, the inset to the right displays the magnified version of the boxed region of the larger micrograph. Black arrowheads point to heterophils (pink cytoplasm, multilobular darkly stained nucleus). Bar, 100 μm. In panel C (MxiM mutant), the inset to the right displays a magnified version of the boxed region of the larger micrograph. Arrowheads point to heterophils. Bar, 100 μm.

Interestingly, the *icsA* mutant led to an accumulation of heterophils (innate immune cells that are the rabbit equivalent of neutrophils) in the colon that was not observed in animals infected with the WT strain (Fig. 6). Thus, IcsA may contribute to immune evasion by limiting the recruitment of innate immune cells. The *mxiM* mutant also recruited more heterophils to the lamina propria and epithelial cell layer than the WT strain (Fig. 6A and C). Unlike the Δ*icsA* and Δ*mxiM* strains, the plasmidless strain did not recruit heterophils in the colon. Thus, both IcsA and T3SS appear to antagonize heterophil recruitment, perhaps by facilitating pathogen invasion. However, the absence of heterophil influx in the plasmidless strain challenges this hypothesis and suggests that another plasmid-encoded factor can counteract the actions of IcsA and/or the T3SS in blocking heterophil infiltration.

**FIG 7:**
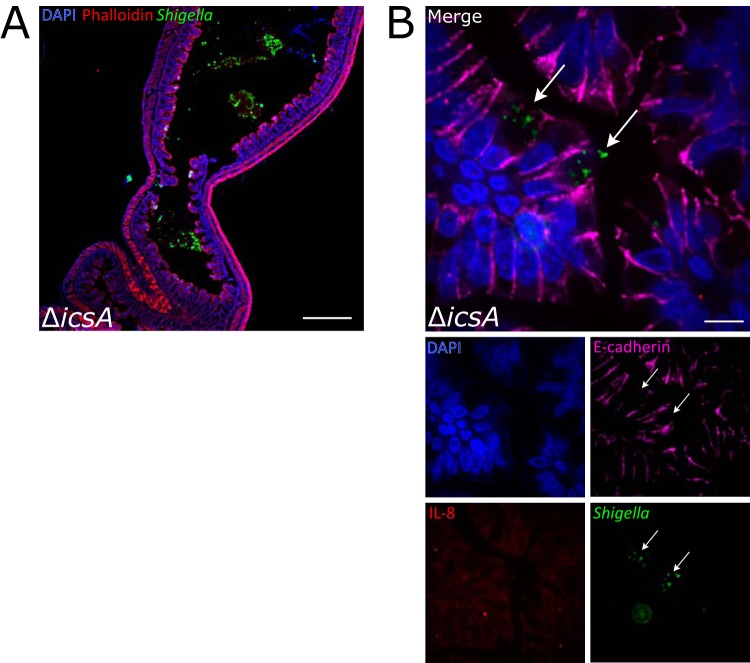
Intestinal localization and IL-8 transcripts in colons from animals infected with an *icsA* mutant. (A) Immunofluorescence micrograph of Δ*icsA* in colonic tissue of infected rabbits 36 hpi. DAPI (blue), FITC-conjugated anti-*Shigella* antibody (green), and phalloidin-Alexa Fluor 568 (red) are shown. Bar, 500 μm. (B) Immunofluorescence micrograph of sections stained with a RNAscope probe to rabbit IL-8 (red) and antibodies to *Shigella* (green) and E-cadherin (magenta), and DAPI (blue). The bottom panels depict channels of the merged image. Arrows point to multiple *icsA* bacteria in the cytoplasm of two infected cells. Bar, 10 μm.

Since colonic pathology was altered in the mutant strains, we investigated the intestinal localization and IL-8 production induced by the mutants. All three of the mutant strains were found almost exclusively in the lumen of the colon (Fig. 7A and Fig. S4); in contrast to the WT strain (Fig. 3), it was difficult to detect infection foci in the epithelial cell layer in animals infected with mutant strains (Fig. 7). The *icsA* mutant was occasionally observed inside epithelial cells (Fig. 7B), but larger foci were not detected. As expected, we observed very few cells expressing IL-8 mRNA in the colons of rabbits infected with any of the three mutant S. flexneri strains (Fig. 4D and 7B), supporting the idea that induction of IL-8 expression requires S. flexneri invasion of the epithelial cell layer in this model.

10.1128/mBio.03105-19.4 FIG S4 Localization of S. flexneri mutants in infected infant rabbits. (A to C) Immunofluorescence micrographs of S. flexneri mutants in colonic tissue of infected rabbits 36 hpi. (A) Inset and white arrows show individual S. flexneri Δ*icsA* cells closely associated with the colonic epithelium. Blue, DAPI; green, FITC-conjugated anti-*Shigella* antibody; red, phalloidin-Alexa Fluor 568. Scale bars, 500 μm (A to C). Download FIG S4, EPS file, 2.8 MB. Copyright © 2020 Kuehl et al. 2020 Kuehl et al. This content is distributed under the terms of the Creative Commons Attribution 4.0 International license.

## DISCUSSION

Small animal models of shigellosis that rely on the oral route of infection have been lacking. Here, we found that orogastric inoculation of 2- to 3-day-old infant rabbits with S. flexneri led to a diarrheal disease and colonic pathology reminiscent of some aspects of human disease. Fasting animals prior to inoculation reduced the variability in infection outcomes, but not all inoculated animals developed disease. The pathogen robustly colonized the colon, where the organism was found primarily in the lumen; however, prominent infection foci were also observed within the colonic epithelium. Robust S. flexneri intestinal colonization, invasion of the colonic epithelium, and colonic epithelial sloughing required IcsA and the T3SS, which are both canonical S. flexneri virulence factors. Despite the reduced intestinal colonization of the *icsA* and *mxiM* mutants, these strains elicited more pronounced colonic inflammation (characterized by infiltration of heterophils) than the WT strain did. IL-8 expression, detected with *in situ* mRNA labeling, was higher in animals infected with the WT versus the mutant strains, suggesting that epithelial invasion promotes expression of this chemokine. Interestingly, IL-8 expression was greater in uninfected cells near infected epithelial cells than in infected epithelial cells themselves. Collectively, our findings suggest that oral infection of infant rabbits offers a useful experimental model for investigations of the pathogenesis of shigellosis.

Fasted animals developed disease more frequently and had elevated intestinal colonization compared to animals who fed *ad libidum* prior to inoculation. The presence of inhibitory substances in milk, such as lactoferrin, which degrades components of the *Shigella* T3SS apparatus (39), may limit bacterial establishment in the intestine but have less potent effects once colonization is established. Mean colonic colonization was higher in animals that developed disease than those that did not (see Fig. S1A in the supplemental material). However, high bacterial burdens are not the only factor predictive of disease; several animals with high pathogen burdens did not exhibit signs of disease (Fig. S1A). Also, initial rabbit body weights did not strongly influence clinical outcomes (Fig. S1C). Several additional factors likely modulate *Shigella* colonization and disease manifestation in infant rabbits. For example, variations in the intestinal microbiota of the infant rabbits may limit or potentiate S. flexneri virulence and/or colonization, as described for infections caused by other enteric pathogens, including Clostridium difficile (60), L. monocytogenes (61), and V. cholerae (62). Differences in dam feeding patterns also likely influence colonization and disease outcomes. Further elucidation of factors that modulate outcomes will be valuable to improve this model because they may point to ways to elevate the fraction of animals that develop clinical signs of infection.

A high inoculum dose (10^9^ CFU) was required to achieve reliable disease development following oral inoculation of 2- to 3-day-old infant rabbits. Animals inoculated with lower doses (e.g., 10^7^ CFU) of S. flexneri developed disease and robust intestinal colonization at lower frequencies. Interestingly, even in oral nonhuman primate models, the standard inoculum dose (10^10^ CFU) to ensure consistent development of disease (63, 64) is orders of magnitude greater than the dose used in human challenge studies (typically 10^3^ to 10^4^ CFU) (37, 65, 66). The reasons accounting for these marked differences in infectious doses warrant further exploration. It is unlikely that older rabbits infected via the oral route will be susceptible to colonization and disease, since our findings with other pathogens (35) suggest that infant rabbits become resistant to oral inoculation with enteric pathogens when they are ∼5 days old.

In human infections, *Shigella* causes colonic pathology characterized by an acute inflammatory response with mucosal ulceration and erosions, neutrophil infiltration, congestion, and hemorrhage (8, 9). In the oral infant rabbit model, the WT strain caused edema and sloughing of epithelial cells in the colon, but we did not observe recruitment of heterophils, suggesting that colonic pathology is not primarily attributable to an acute inflammatory response characterized by heterophil infiltration. Instead, the pathology may be driven by invasion and replication of the pathogen within colonic epithelial cells. Orogastric inoculation of infant rabbits with EHEC induces heterophil infiltration in the colon (34), indicating that these animals are capable of mounting an acute inflammatory response in this organ.

The marked colonization defect of the Δ*icsA* strain, matching that observed for the Δ*mxiM* (T3SS-deficient) and plasmidless strains, was unexpected. It seems unlikely the Δ*icsA* mutant’s colonization defect is entirely attributable to the mutant’s deficiency in cell-to-cell spreading. Brotcke Zumsteg et al. found that IcsA can also serve as an adhesin (67). Since distinct regions of IcsA are required for its adhesive versus cell spreading activities (67), it may be possible to genetically dissect which of these IcsA functions plays a dominant role in colonization, using S. flexneri strains producing mutant versions of IcsA. Passage of the pathogen through the upper gastrointestinal tract may be required to reveal IcsA’s adhesive activity, because a Δ*icsA* strain had only a modest colonization defect after intrarectal instillation (10). It was also surprising that the animals infected with the Δ*icsA* strain recruited heterophils to the colon despite little induction of IL-8 expression. These observations suggest that there are additional factors contributing to heterophil recruitment to the rabbit colon. Moreover, since there is minimal heterophil recruitment in animals infected with the WT strain, IcsA-mediated pathogen adherence to colonic epithelial cells (and potentially concomitant increased invasion) may increase delivery of T3SS effectors into host cells, thereby repressing a host-derived heterophil recruitment factor.

Our attempts to utilize TIS to identify novel genetic loci contributing to S. flexneri colonization in the infant rabbit intestine were stymied by a narrow infection bottleneck. The tight bottleneck leads to large, random losses of genetic diversity of the input library. The underlying causes of *in vivo* bottlenecks vary and may include stomach acidity, host innate immune defenses, such as antimicrobial peptides, the number of available niches in the intestine, and competition with the endogenous commensal microbiota (68). Modifications to either the inoculation protocol or library generation could facilitate future *in vivo* TIS screens. For example, the diversity of the inoculum could be reduced by generating a defined library of transposon mutants with only one or two mutants per gene (e.g., as has been done in Edwardsiella piscicida [69]). Regarding the infection protocol, it is possible that the fraction of the inoculum that initially seeds and colonizes the intestine could be elevated by reducing the number of commensal organisms in the intestine that may compete for a niche similar to that occupied by S. flexneri. Similar strategies have been utilized to facilitate studies of other enteric pathogens (61, 70).

The intrarectal infant rabbit model of shigellosis reported by Yum et al. (10) has some beneficial features compared to the oral infection model. Using this route, Yum et al. reported that all animals developed bloody diarrhea and colonic pathology that included substantial recruitment of heterophils (10). As noted above, for unknown reasons, oral inoculation of WT S. flexneri did not lead to heterophil recruitment to sites of damage in the colon. An additional difference is that intrarectal instillation of a Δ*icsA* mutant led to induction of cytokine expression, heterophil recruitment, and only slightly reduced colonization of the strain, whereas following oral inoculation, a Δ*icsA* S. flexneri exhibited a marked colonization defect and did not induce IL-8 mRNA expression. Additional studies are required to elucidate the reasons that account for the differential importance of IcsA in these models. While some features of the intrarectal model are attractive, Yum et al. used 2-week-old rabbits that were carefully hand reared in an animal facility from birth using a complex protocol that may prove difficult for others to adopt (10). In addition to the physiologic route of infection, the oral infant rabbit model requires far less specialized animal husbandry than the intrarectal model and may therefore prove more accessible.

In summary, oral inoculation of infant rabbits with *Shigella* provides a feasible small animal model to study the pathogenesis of this globally important enteric pathogen. The model should also be useful to test new therapeutics for shigellosis, an issue of increasing importance given the development of *Shigella* strains with increasing resistance to multiple antibiotics (71–74).

## MATERIALS AND METHODS

### Bacterial strains and growth.

Bacterial strains are listed in Table S2 in the supplemental material. S. flexneri bacteria were routinely grown aerobically in Miller lysogeny broth (LB) or LB agar at 30°C or 37°C. Antibiotics, when used, were included at the following concentrations: streptomycin (Sm), 200 μg/ml; kanamycin (Km), 50 μg/ml; carbenicillin (Carb), 100 μg/ml; and chloramphenicol (Cm), 10 μg/ml. To check for the presence of the virulence plasmid, bacteria were grown on media with Congo red added at 0.1% (wt/vol).

10.1128/mBio.03105-19.6 TABLE S2 Strains, plasmids, and oligonucleotides. Download Table S2, XLSX file, 0.01 MB. Copyright © 2020 Kuehl et al. 2020 Kuehl et al. This content is distributed under the terms of the Creative Commons Attribution 4.0 International license.

E. coli bacteria were routinely grown in LB media or agar. Antibiotics were used at the same concentrations as S. flexneri except for Cm, which was 30 μg/ml. When required, diaminopimelic acid (DAP) was added at a concentration of 0.3 mM.

### Strain construction.

S. flexneri 2a strains 2457T and BS103 (a derivative lacking the virulence plasmid) were gifts of Marcia Goldberg. A spontaneous streptomycin-resistant strain of S. flexneri 2a strain 2457T was generated by plating overnight LB cultures of S. flexneri 2a 2457T on LB plates containing 1,000 μg/ml Sm and identifying Sm-resistant (Sm^r^) strains that grew as well as the parent strain. The Sm^r^ strain was used as the wild-type (WT) strain for all subsequent experiments, including animal experiments and construction of mutant strains. Primers (Table S2) were used to amplify the *rpsL* gene in the strain, and Sanger sequencing was performed to determine the nature of the mutation resulting in streptomycin resistance. The streptomycin resistance allele was transferred from the Sm^r^ wild-type strain into strain BS103 by P1 transduction, yielding a Sm^r^ plasmidless strain.

Single gene deletion mutants were generated in the WT Sm^r^ strain using the lambda red recombination method, as previously described (75). Resistance cassettes used in the process were amplified from pKD3 (Cm). Mutations generated by lambda red were moved into a clean genetic background by transferring the mutation to the Sm^r^ wild-type strain via P1 transduction. Subsequently, antibiotic resistance cassettes were removed via FLP-mediated recombination using pCP20. Retention of the virulence plasmid throughout P1 transduction of the mutation into the parental WT Sm^r^ strain was monitored by plating bacterial mutants on LB plus Congo red to identify red colonies and by performing multiplex PCR for various genes spread across the virulence plasmid (primers are listed in Table S2).

### Animal experiments.

Rabbit experiments were conducted according to the recommendations of the National Institutes of Health Guide for the Care and Use of Laboratory Animals, the Animal Welfare Act of the U.S. Department of Agriculture, and the Brigham and Women’s Hospital Committee on Animals, as outlined in Institutional Animal Care and Use Compliance protocol 2016N000334 and Animal Welfare Assurance of Compliance A4752-01.

Litters of 2- to 3-day-old New Zealand White infant rabbits with lactating adult female (dam) obtained from a commercial breeder (Charles River, Canada, or Pine Acres Rabbitry Farm & Research Facility, Norton, MA) were used for animal experiments.

Infant rabbits were administered a subcutaneous injection of Zantac (ranitidine hydrochloride; 50 mg/kg of body weight; GlaxoSmithKline) 3 h prior to inoculation with the wild type (Sm^r^) or isogenic mutants. We attempted to utilize a bicarbonate solution to administer bacteria but found that S. flexneri does not survive when resuspended in a sodium bicarbonate solution. For initial experiments, a day after arrival, infant rabbits were orogastrically inoculated with 1e9 CFU of log-phase S. flexneri suspended in LB. To prepare the inoculum, an overnight bacterial culture grown at 30°C was diluted 1:100 and grown at 37°C for 3 h. The bacteria were subsequently pelleted and resuspended in fresh LB to a final concentration of 2e9 CFU/ml. Rabbits were orogastrically inoculated using a PE50 catheter (Becton Dickson) with 0.5 ml of inoculum (1e9 CFU total). In later experiments, infant rabbits were first separated from the dam for 24 h prior to inoculation, after which they were immediately returned to the dam for the remainder of the experiment.

The infant rabbits were then observed for 36 to 40 h postinoculation and then euthanized via isoflurane inhalation and subsequent intracardiac injection of 6 mEq KCl at the end of the experiment or when they became moribund. Animals were checked for signs of disease every 10 to 12 h. Body weight and body temperature measurements were made 1 or 2 times daily until the end of the experiment. Body temperature was measured with a digital temporal thermometer (Exergen) and assessed on the infant rabbit chest, in between the front legs. Temperatures reported in Fig. 1E are the final temperatures prior to euthanasia, and change in body weight in Fig. 1F is a comparison of the final to initial body weight. Diarrhea was scored as follows: no diarrhea (solid feces, no adherent stool on paw region) or diarrhea (liquid fecal material adhering to hind paw region). Animal experiments with isogenic mutants were always conducted with littermate controls infected with the WT Sm^r^ strain to control litter variation.

At necropsy, the intestine from the duodenum to rectum was dissected and divided into separate anatomical sections (small intestine, colon) as previously described (54, 76). Pieces (1 to 2 cm) of each anatomical section were used for measurements of tissue bacterial burden. Tissue samples were placed in 1× phosphate-buffered saline (PBS) with two stainless steel beads and homogenized with a bead beater (BioSpec Products Inc.). Serial dilutions were made using 1× PBS and plated on LB plus Sm (LB+Sm) medium for enumeration of bacterial CFU. For processing of tissue for microscopy, 1- to 2-cm pieces of the tissue adjacent to the piece taken for enumeration of bacterial CFU were submerged in either 4% paraformaldehyde (PFA) for frozen sections or 10% neutral buffered formalin (NBF) for paraffin sections.

For gentamicin tissue assays, a 1- to 2-cm portion of the colon was cut open longitudinally and washed in 1× PBS to remove luminal contents and then incubated in 1 ml of 1× Dulbecco modified Eagle medium (DMEM) with 100 μg/ml gentamicin for 1 h at room temperature. The tissue was subsequently washed three times with 20× volumes of 1× PBS for 30 min with shaking. The tissue was then homogenized, and serial dilutions were plated on LB+Sm medium for enumeration of bacterial burden.

For Tn-seq experiments, aliquots of the transposon library were thawed and aerobically cultured in LB for 3 h. The bacteria were pelleted and resuspended in fresh LB to a final concentration of 1e9 CFU per 0.5-ml inoculum. A sample of the input library (1e10 CFU) was plated on a large LB+Sm+Km plate (245 cm^2^; Corning). Bacterial burdens in infected rabbit tissues were determined by plating serial dilutions on LB+Sm+Km plates. The entire colon was homogenized and plated onto a large LB+Sm+Km plate to recover transposon mutants that survived in the colon. Bacteria on large plates were grown for ∼20 to 22 h at 30°C and scraped off with ∼10 ml fresh LB, and ∼1-ml aliquots were pelleted. The pellets were frozen at –80°C prior to genomic DNA extraction for Tn-seq library construction.

Data from animal experiments were analyzed in Prism (version [ver.] 8; GraphPad). The Mann-Whitney U test or the Kruskal-Wallis test with Dunn’s posttest for multiple comparisons were used to compare the tissue bacterial burdens. A Fisher’s exact test was used to compare the proportion of rabbits that developed diarrhea after infection with various bacterial strains.

### Immunofluorescence microscopy.

Immunofluorescence images were analyzed from 20 wild-type rabbits and at least 4 rabbits infected with each of the various mutant bacterial strains, or uninfected rabbits; 2 or 3 colon sections per rabbit were examined. Tissue samples used for immunofluorescence were fixed in 4% PFA, and subsequently stored in 30% sucrose prior to embedding in a 1:2.5 mixture of OCT (Tissue-Tek) to 30% sucrose and stored at –80°C, as previously described (35). Frozen sections were cut at a thickness of 10 μm using a cryotome (catalog no. CM1860UV; Leica). Sections were first blocked with 5% bovine serum albumin (BSA) in PBS for 1 h. Sections were stained overnight at 4°C with a primary antibody, diluted in PBS with 0.5% BSA and 0.5% Triton X-100, anti-*Shigella* labeled with fluorescein isothiocyanate (FITC) (1/1,000) (catalog no. 0903, Virostat), and anti-E-cadherin (1:100) (catalog no. 610181; BD Biosciences). After washing with 1× PBS containing 0.5% Tween 20 (PBST), sections were incubated with Alexa Fluor 647 phalloidin (1/1000; Invitrogen) for 1 h at room temperature, washed, and stained for 5 min with 4′,6-diamidino-2-phenylindole (DAPI) at 2 μg/ml for 5 min, and covered with ProLong Diamond or Glass Antifade (Invitrogen) mounting medium. Slides were imaged using a Nikon Ti Eclipse equipped with a spinning disk confocal scanner unit (Yokogawa CSU-Xu1) and electron-multiplying charge-coupled-device (EMCCD) (Andor iXon3) camera, or with a scientific complementary metal-oxide-semiconductor (sCMOS) camera (Andor Zyla) for wide-field microscopy.

### Histopathology.

Tissue samples used for histopathology analysis were fixed in 10% NBF and subsequently stored in 70% ethanol prior to being embedded in paraffin as previously described (36). Formalin-fixed, paraffin-embedded (FFPE) sections were made at a thickness of 5 μm. Sections were stained with hematoxylin and eosin (H&E). Slides were assessed for various measures of pathology, e.g., heterophil infiltration, edema, epithelial sloughing, hemorrhage, by a pathologist blind to the tissue origin. Semiquantitative scores for heterophil infiltration were as follows: 0 for no heterophils observed; 1 for rare heterophils; 2 for few heterophils; 3 for many heterophils; 4 for abundant heterophils. Bright-field micrographs were collected using an Olympus VS120 system.

### *In situ* RNA hybridization.

Freshly cut FFPE sections (5 μm) were made of the indicated anatomical sections and stored with desiccants at 4°C. Subsequently, sections were processed and analyzed using the RNAscope Multiplex Fluorescent v2 Assay (Advanced Cell Diagnostics USA-ACDbio) combined with immunofluorescence. Briefly, sections were processed following ACDbio recommendations for FFPE sample preparation and pretreatment using 15-min target retrieval and 25-min Protease Plus digestion using the RNAscope HybEZ oven for all incubations. An RNAscope C1 probe (OcIL8) to rabbit chemokine (C-X-C motif) ligand 8 (CXCL8) was developed and used to stain intestinal sections for CXCL8 mRNA expression. The C1 probe was detected with Opal 570 dye (Akoya Biosciences) diluted 1:1,000 in Multiplex TSA buffer (ACDbio). Sections were also stained with DAPI (2 μg/ml), anti-*Shigella* labeled with FITC (1/1,000; Virostat), and anti-mouse E-cadherin (1/100) (catalog no. 610181, BD Biosciences). Slides were imaged using a Nikon Ti Eclipse equipped with a spinning disk confocal scanner unit (Yokogawa CSU-Xu1) and EMCCD (Andor iXon3) camera for high-magnification images. Slides were imaged using a wide-field Zeiss Axioplan 2 microscope through the MetaMorph imaging system for RNAscope signal quantification.

### Quantitative image analysis.

Images of mid-colon tissue sections stained with RNAscope OcIL8, DAPI, and FITC-conjugated anti-*Shigella* antibody were acquired and analyzed using the MetaMorph (7.1.4.0) application. Briefly, tiled 10× images covering the entire length of the tissue section were collected using multidimensional acquisition. For analysis of the percentage of IL-8 mRNA-expressing cells that were adjacent to infected cells, we analyzed 86 foci of infection at 100× magnification. Exclusive threshold values were set for the DAPI channel or the rhodamine channel independently and applied to all images in the data set. The threshold values for DAPI or rhodamine were used to create a binary mask of each image. The total area under the binary mask was recorded and used to calculate the percentage of total tissue (DAPI area under mask) expressing CXCL8 mRNA (rhodamine area under mask) by dividing the values for rhodamine area by the DAPI area for each image. Percentages were graphed using Prism version 8 (GraphPad).

### Transposon library construction and analysis.

A transposon library was constructed in S. flexneri 2a 2457T Sm^r^ (WT Sm^r^) using pSC189 (77) by previously described protocols (54, 78) with additional modifications. Briefly, E. coli strain MFD*pir* (79) was transformed with pSC189. Conjugation was performed between WT Sm^r^ and MFD*pir* pSC189. Overnight LB cultures of WT Sm^r^ (grown at 30°C) and MFD*pir* (grown at 37°C) were mixed and spotted onto 0.45-μm filters on LB+DAP agar plates. The conjugation reaction was allowed to proceed for 2 h at 30°C. Subsequently, the bacterial mixtures were resuspended in LB and spread across four 245-cm^2^ LB+Sm+Km square plates to generate single separate colonies for a transposon library. The square plates were grown at 30°C for 20 h. The colonies that formed (∼800,000 total) were washed off with LB (8 ml per plate), and the bacteria from two plates were combined. Two separate 1-ml aliquots of the two combined mixtures were used to start two 100-ml LB+Km liquid cultures. The cultures were grown aerobically at 30°C with shaking for 3 h. For each flask, the bacteria were pelleted and resuspended in a small amount of LB before being spread across two 245-cm^2^ LB+Sm+Km square plates and grown at 30°C for 20 h. The resulting bacteria on the plate were washed off with LB and resuspended. The optical density (OD) was adjusted to 10 with LB and glycerol so that the final concentration of glycerol was 25%. One-milliliter LB+glycerol aliquots were stored at –80°C for later experiments. In addition, 1-ml aliquots were also pelleted to generate bacterial pellets to serve as sources of genomic DNA for the initial characterization of the transposon library. The pellets were stored at –80°C prior to genomic DNA extraction for Tn-seq library construction.

Tn-seq library construction and data analysis were performed as previously described (54, 55, 80); briefly, genomic DNA was extracted, transposon junctions were amplified, sequencing was performed on an Illumina MiSeq instrument, and data were analyzed using a modified ARTIST pipeline (54, 55). Sequence reads were mapped onto the S. flexneri 2a strain 2457T chromosome (RefSeq accession no. NC_004741.1) and S. flexneri 2a strain 301 virulence plasmid (RefSeq accession no. NC_004851.1). Reads at each TA site were tallied.
