# Supplementary figures and images for: An Oral Inoculation Infant Rabbit Model for Shigella Infection
Source: mBio. 2020 Jan 21;11(1):e03105-19. doi: 10.1128/mBio.03105-19 (PMC6974573; doi:10.1128/mBio.03105-19)

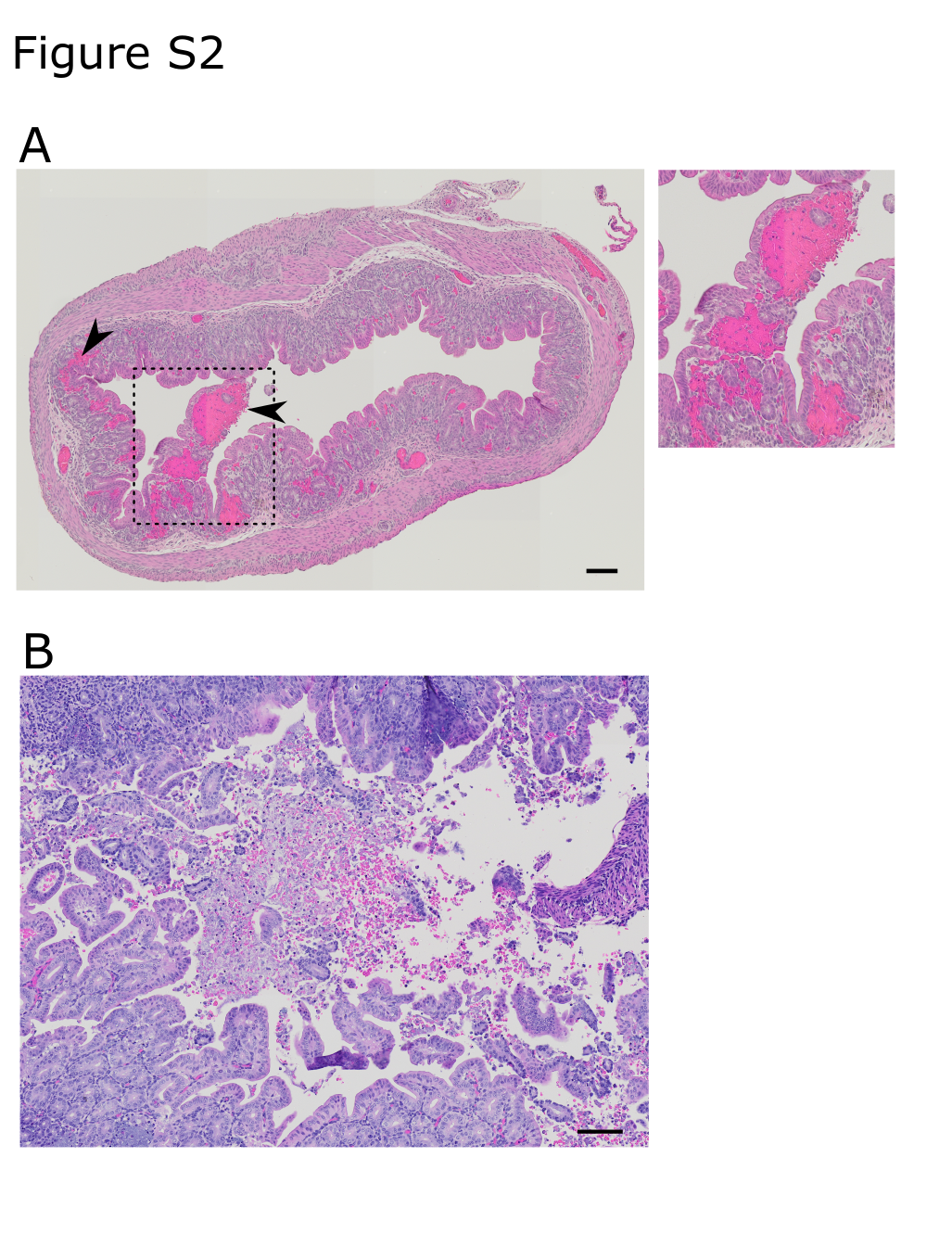

Supplement: FIG S2 [file mBio.03105-19-sf002.tif]

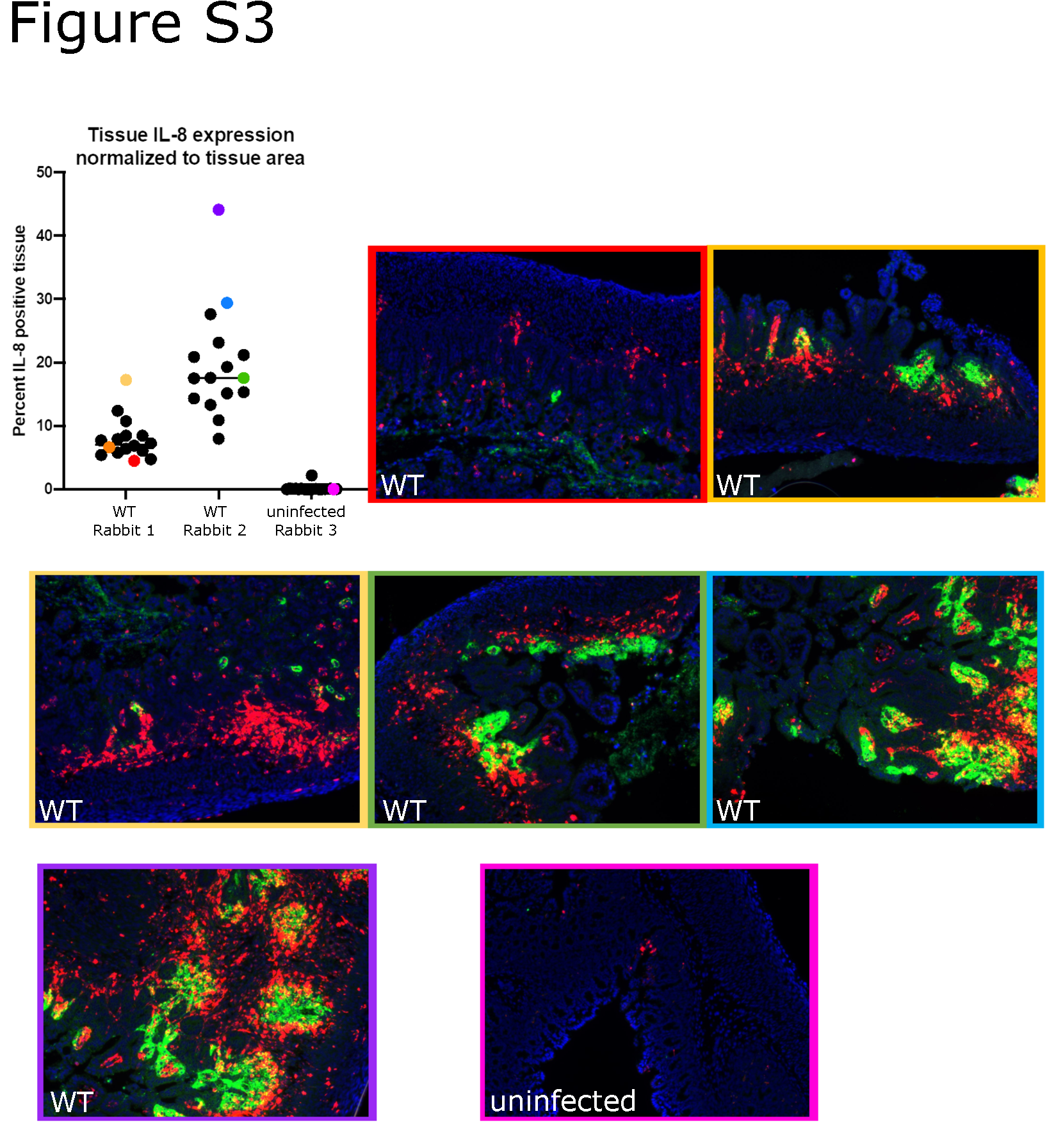

Supplement: FIG S3 [file mBio.03105-19-sf003.tif]
